# Supplementary figures and images for: Effects of mild-to-moderate sensorineural hearing loss and signal amplification on vocal emotion recognition in middle-aged–older individuals
Source: PLoS One. 2025 Jul 16;20(7):e0322867. doi: 10.1371/journal.pone.0322867 (PMC12266405; doi:10.1371/journal.pone.0322867)

**SI Appendix**

Results from nominal regression analyses with different emotions as references.


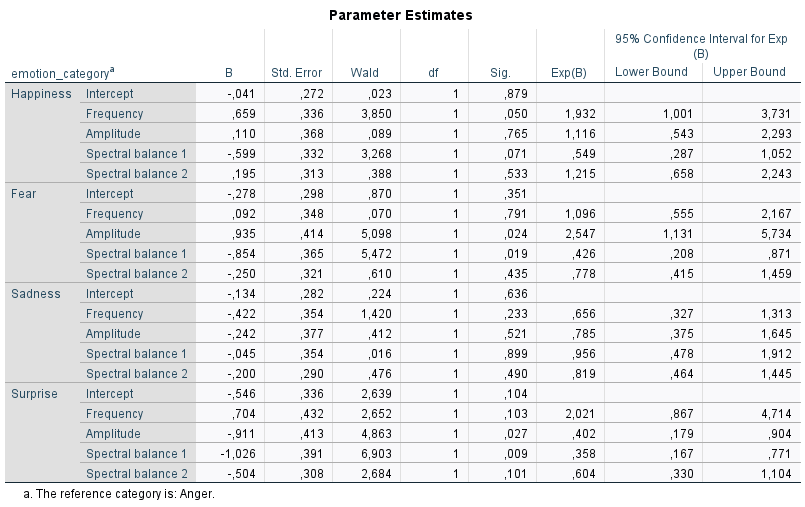

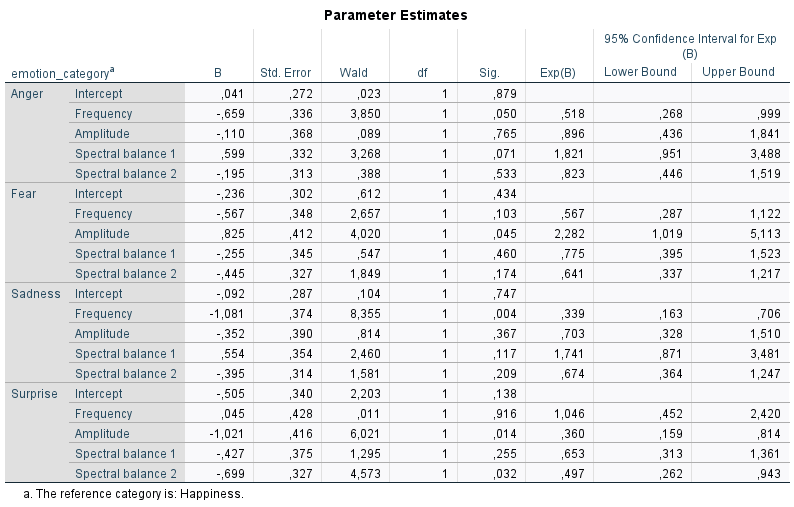

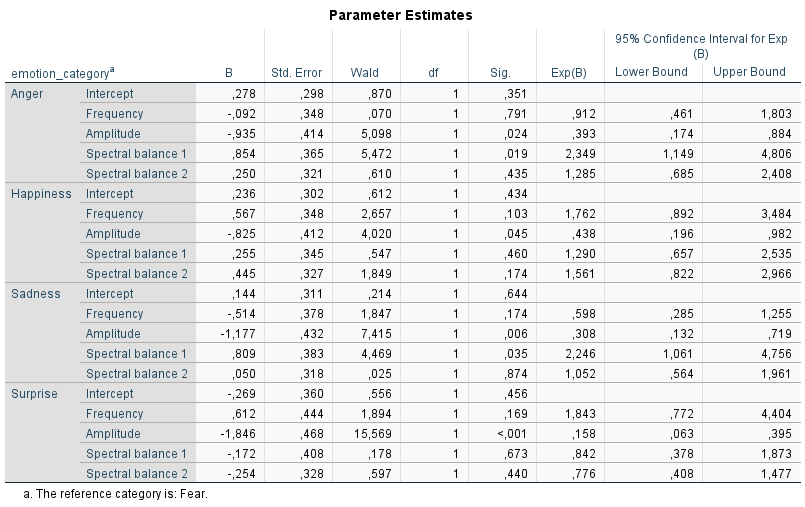

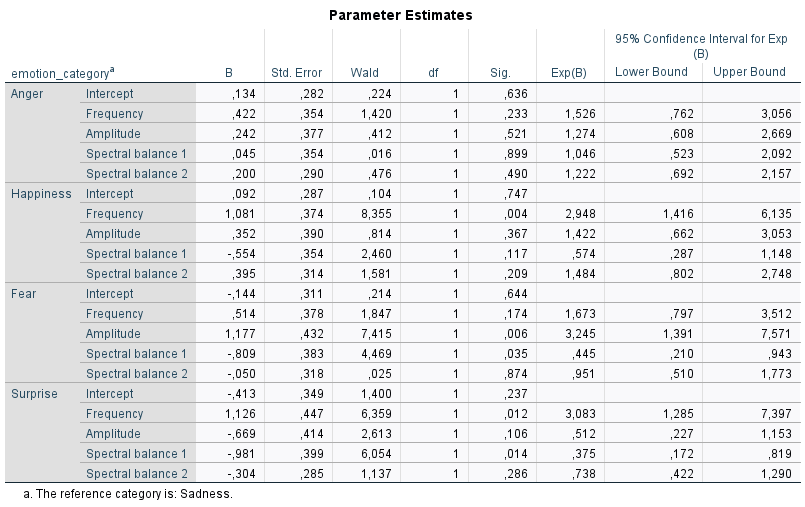

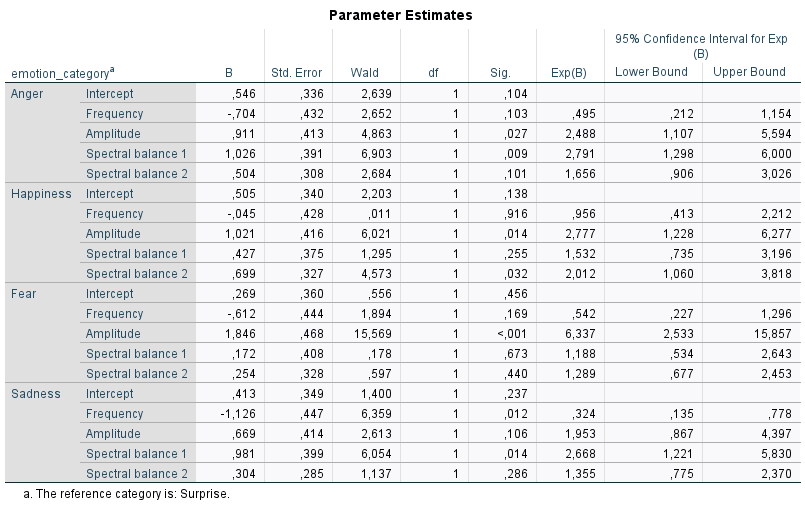

Supplement: S1 Table — (DOCX) [file pone.0322867.s002.docx]
