# Supplementary material for: Effects of mild-to-moderate sensorineural hearing loss and signal amplification on vocal emotion recognition in middle-aged–older individuals
Source: PLoS One. 2025 Jul 16;20(7):e0322867. doi: 10.1371/journal.pone.0322867 (PMC12266405; doi:10.1371/journal.pone.0322867)
Supplement: Attachment — Submitted filename: My Comments.docx [file pone.0322867.s003.docx]

**Abstract:**

The abstract clearly defines the importance of the research subject, highlighting the deficiency in comprehension about the impact of hearing loss on emotion detection, notwithstanding progress in hearing aid technology. Nonetheless, below is an evaluation of its areas for enhancement:

1. It may be beneficial to briefly define phrases such as "linear amplification" for readers unfamiliar with audiological terminology.
2. Incorporating precise statistics or effect sizes pertaining to enhancements in emotion recognition could enrich the findings and their importance.

**Introduction:**

The introduction offers a thorough review of the context and importance of the study on vocal emotion identification in individuals with hearing impairment. Here are recommendations for enhancement:

1. The flow could be improved with appropriate section organization. For instance, categorizing the sections on “Vocal Emotion Recognition” and “Effects of Hearing Loss” with specific subheadings could enhance readability, my suggestion is to add these subheadings to the introduction text accordingly, such as under “Vocal Emotion Recognition” section

- Importance of Vocal Emotion Recognition
- Acoustic Characteristics of Vocal Emotions
- The impact of auditory impairment on the perception of voice emotions

Furthermore, add this subheading under “Effects of Hearing Loss” section

- Prevalence of Hearing Loss

2. Now, the introduction does not include information regarding the demographics of the participants in the earlier studies, it is important to briefly explain (in one paragraph) the demographic data (other age groups) of individuals who participated in prior studies. This will help to contextualize the findings and ensure that they are applicable to a wider population.
4. There are some typo errors e.g., “different lower--level features”, “not yielded entirelyconsistent results”, (see for example 5-8 and 16-18),. hearing loss that inloves involves, Thisis likely related to, commonly usedto characterize.

**Materials and Methods:**

The following are a few recommendations for enhancing the Methods section of your document:

2.1 **Participants**

1. Revise Inclusion/Exclusion Criteria: Clearly differentiate between inclusion and exclusion criteria
2. The p-value should be written as p<0.001 rather than p<.001
3. The sociodemographic data should be presented in the results section.
4. Table 1 Description: Summarize Table 1 with a sentence that emphasizes the significant demographic differences before its presentation. Also, the table needs to be presented in the results section.

**Results:**

The results section is comprehensive and provides extensive information of the findings. Here are some aspects for enhancement to contemplate for the article review:

1. The structure of the result might be improved by having clearer **subsections**, for example, Descriptive Data, Recognition Accuracy, Effects of Hearing Loss, and "Effects of Amplification, with titles for each **subsection**. The reader would be better able to navigate the findings in a methodical manner if this was done.
2. In Tables 3a and 3b, the authors may merge the columns of acoustic feature and acoustic parameter
3. All figures need improvement regarding their quality.

**Discussion:**

The research effectively addresses a gap in the current state of knowledge concerning the influence of linear amplification on emotion recognition. The discussion is comprehensive, confronting the limitations and relating the findings to existing research in a transparent manner. However, the authors may suggest:

1. Future research should evaluate sensorineural hearing loss based on clinical diagnosis rather than self-reporting.
2. Table 5 was not mentioned or referred to it in the discussion section.

**Conclusion:**

The conclusion that authors have composed is coherent and highlights the principal conclusions of your study; but it would be advantageous to adopt a more official tone, implement a little rearrangement for enhanced coherence, and incorporate a more prospective outlook. Here is a polished iteration:

1. Restructured the principal points to facilitate a more seamless transition from overarching findings to particular results and implications.

2. Incorporated a request for further research, enhancing the conclusion's forward-thinking nature and highlighting potential avenues that your work may inspire.
