# Supplementary material for: Effects of mild-to-moderate sensorineural hearing loss and signal amplification on vocal emotion recognition in middle-aged–older individuals
Source: PLoS One. 2025 Jul 16;20(7):e0322867. doi: 10.1371/journal.pone.0322867 (PMC12266405; doi:10.1371/journal.pone.0322867)
Supplement: Attachment — Submitted filename: Response to Reviewers.docx [file pone.0322867.s004.docx]

Dear Editor,

We thank you and the reviewers for the positive feedback and the suggestions and for giving us the ability to revise the manuscript “Effects of mild-to-moderate sensorineural hearing loss and signal amplification on vocal emotion recognition in middle-aged–older individuals“. We have made considerable changes to the manuscript in response to the reviewers’ comments. The main changes we have made are:

Although we were under the impression that the Introduction and Methods section should not be changed in a Stage 2 (see Rule 9 in https://journals.plos.org/ploscompbiol/article?id=10.1371/journal.pcbi.1010571), we have revised the introduction based on the recommendations from the reveiwers and the editor. Especially we have made general clarifications, included more about emotion-acoustics and made changes to the structure (as suggested by the editor).

In methods, we have added some new headings, clarified the inclusion and exclusion criteria, the description of the study material, the use of Rosenthal index and the linear amplification method. There is one difference in methods compared with what was presented in the Stage 1 article and we have now highlighted this and motivated why (audiograms could not be delivered from medical journals, and instead we included tone audiograms also for individuals with hearing loss in our testing procedure). We have also added descriptions of how distinctiveness of emotions in sentences is described and descriptions of the linear amplification [ED 2-3].

Regarding the predictions in the introduction, we have changed the phrasing in predictions 3 and 4 from “salient” to “distinct”, as the original phrasing might unintendedly imply that we would directly analyze associations between frequency-related acoustic parameters and recognition accuracy. This is not the case. When asked before the stage 1 publication how we would relate acoustics to the behavioural data we responded that this would be done in a qualitative descriptive manner.

- In the results, we have added a description of demographics (moved from the methods section) and results from analyses of acoustic differences between emotions and acoustic distances, with new headings. The rationale for dealing with missing data is also addressed. We also added results describing acoustic differences between emotions in sentences [ED 4][ED 4]
- The discussion has been revised to have a clearer connection between the findings and our hypotheses/predictions. There is also a new section addressing potential implications for clinical practice and future research directions. [ED 5-6]
- Finally, we have updated numbering of figures and tables, proofread for grammatical errors and typos, changed filenames according to PLOS ONE’s style requirements, and reviewed the reference list. [ED 7-12].

Sincerely yours,

The authors

**Academic editor**

***Introduction***

**Comment 1**. “Consider providing a brief overview of the existing literature on vocal emotion recognition in individuals with hearing loss. This context will strengthen the rationale for your study”

Response:

Thank you for your suggestion. In response, we have included additional text (pages 6-7 ) to provide an overview of the existing literature on vocal emotion recognition in individuals with hearing loss. Furthermore, we have made additional changes to the introduction. These include structural changes, detailed descriptions of previous studies on the acoustic characteristics of different emotion categories, and a rationale for examining the effects of linear amplification.

***Methods***

**Comment 2.** “”Please clarify the criteria for participant selection. A more detailed description of inclusion/exclusion criteria would enhance the reproducibility of your study.”

Response:

We have clarified the criteria for participant selection by including more detailed descriptions and dividing the text into separate sections for inclusion and exclusion criteria, as suggested by one of the reviewers. (Pages 10 and 11)

**Comment 3**. “It would be beneficial to specify the types of signal amplification used and how they were implemented during the vocal emotion recognition tasks”

Response:

Thank you for this valuable suggestion. We agree that specifying the types of signal amplification and implementation is beneficial. Accordingly, we have added a detailed description on page 13 :

” The stimuli were delivered through AKG K182 headphones, connected to a Supelux HA3D headphone amplifier. In the aided listening conditions, the audio files were preprocessed in MATLAB v. R2022a using an algorithm that implemented the Cambridge formula for linear hearing aids (36), based on each participant’s audiogram. These filtered audio files were then presented to individuals with hearing impairment, simulating the experience of hearing through hearing aids with the specific gain function”

***Results***

**Comment 4.**“Clarifying how missing data were handled would be useful for interpreting the robustness of your results.”

Response:

We agree with the reviewers concern regarding missing data. However, we would like to clarify that there were no instances of missing data in this study. The experimental task was designed to ensure that participants could not refrain from responding unless they chose to terminate the task prematurely. Notably, no participant opted to terminate before completion. We have added a statement about this in the text (page 32):

“No participant aborted any trials in the emotion recognition task, therefore, there is no missing data. “

***Discussion***

**Comment 5.**“In discussing your findings, consider linking them back to the specific hypotheses you outlined in the introduction. This will reinforce the contributions of your work to the field**.”**

Response:

Thank you for this suggestion. We have revised our discussion to more clearly link our findings to the predictions outlined in the introduction. Additionally, to maintain coherence and logial flow, we have reordered the predictions in the introduction. Specifically, prediction number 3 is now presented last, as prediction number 5. This adjustment ensures that the discussion follows a more logical and thematic sequence.

**Comment 6.** “It would be valuable to address potential implications for clinical practice or future research directions based on your findings”

Response:

We agree that addressing potential implication for clinical practice and future research would enhance the value of our text. As our results do not directly say anything about clinical practice, we have allowed ourselves to address potential implications more generally, by putting forth the need to raise awareness among patients and their communication partners about potential difficulties. We also discuss the insufficiency of linear amplification as a solution and suggest that future studies should explore the benefits of vocal emotion recognition training. Additionally, we suggest that future studies should directly compare the effects of linear amplification with those of amplification with compression (which is typically implemented in hearing aids), in controlled experiments.

***Figures and Tables***

**Comment 7.** “Ensure that all figures and tables are clearly labeled and referenced in the text. Consider adding a brief description of what each figure/table illustrates”

Response:

We appreciate the reviewer’s observation. We have identified that table 5 (in the revised version this table is table 9) was not referenced in the text and not described. We have added a description.

***Minor Edits***

**Comment 8.** “Proofread for any grammatical errors or typos to improve the overall professionalism of the report”

Response:

We have thoroughly reviewed the manuscript for grammatical errors and typographical mistakes and have made the necessary corrections to enhance the report.

***Journal requirements***

**Comment 9.** “Please ensure that your manuscript meets PLOS ONE's style requirements, including those for file naming. The PLOS ONE style templates can be found at

<https://journals.plos.org/plosone/s/file?id=wjVg/PLOSOne_formatting_sample_main_body.pdf> and

<https://journals.plos.org/plosone/s/file?id=ba62/PLOSOne_formatting_sample_title_authors_affiliations.pdf>”

Response:

We have updated the file names to comply with the journal’s style requirements.

**Comment 10.** “We noticed you have some minor occurrence of overlapping text with the following previous publication(s), which needs to be addressed: https://journals.plos.org/plosone/article?id=10.1371/journal.pone.0261354. In your revision ensure you cite all your sources (including your own works), and quote or rephrase any duplicated text outside the methods section. Further consideration is dependent on these concerns being addressed”

Response:

We have searched for overlapping text with the stage 1 registered report. The introduction has been substantially revised, so there should no longer be any such overlap.

**Comment 11.** “When completing the data availability statement of the submission form, you indicated that you will make your data available on acceptance. We strongly recommend all authors decide on a data sharing plan before acceptance, as the process can be lengthy and hold up publication timelines. Please note that, though access restrictions are acceptable now, your entire data will need to be made freely accessible if your manuscript is accepted for publication. This policy applies to all data except where public deposition would breach compliance with the protocol approved by your research ethics board. If you are unable to adhere to our open data policy, please kindly revise your statement to explain your reasoning and we will seek the editor's input on an exemption. Please be assured that, once you have provided your new statement, the assessment of your exemption will not hold up the peer review process”

Response:

Data from the experimental task and the acoustic analyses together with scripts and the material used in the study are publicly available on OSF. This sentence has been added to the manuscript:

“The data that support the findings of this study are openly available at the Open Science framework (OSF) at https://osf.io/, Identifier: DOI 10.17605/OSF.IO/V3ANK. Project name: “Vocal emotion identification in middle-aged-to older individuals with sensorineural hearing loss and normal hearing individuals”.

We do not have permission from the review ethics board to share personal information from the online survey. Since this information was only used for checking inclusion and exclusion criteria, it is not needed to evaluate the results of the experimental task.

**Comment 12.** “Please review your reference list to ensure that it is complete and correct. If you have cited papers that have been retracted, please include the rationale for doing so in the manuscript text, or remove these references and replace them with relevant current references. Any changes to the reference list should be mentioned in the rebuttal letter that accompanies your revised manuscript. If you need to cite a retracted article, indicate the article’s retracted status in the References list and also include a citation and full reference for the retraction notice”

Response:

We have reviewed our reference list, and it is complete and correct.

**Reviewer #1**

***General***

**Comment 13.** “It is an rigourus and detailed work.”

Response:

Thank you for this positive assessment.

***Methods***

**Comment 14.** “When are describing the participants you report sociodemographic data, these should be reported on chapter of results”

Response:

We have moved the description of sociodemographic data, including the corresponding table, to the Results section.

**Comment 15.** “Although the manuscript has the title of study design, it is not reported. Instead, the authors describe the procedure for assess the outcome variables”

Response:

Thank you for drawing our attention to this issue. We have made the following changes to address it: The subsection previously titled “*Study design*” has been renamed to “*Procedure*”. We have also added more detailed information regarding the implementation of linear amplification.

***Results***

**Comment 16.** “Table 3a. In the footnote: Note. Number of recordings in total =108; N with expression of anger=24, happiness=25, fear=22, sadness=11, surprise=16. The total of the sum of these values is 98”

Response:

Thank you for pointing out this error. The number of sentences with the expression of sadness was in fact 21, not 11. We have changed from 11 to 21 in the footnote of table 3a (now Table 3).

***Limitation of the study***

**Comment 17.** “You recognized the limitations and discussed these. The main limitiation from my point of view if the groups are not comparable by the age. However, you control it with different analyses. The other important limitation was the power of the achieved sample size. It is a great reason for continuos the study in a further step.”

Response:

We agree that these are significant limitations. It would have been more optimal to have age-matched groups, and we acknowledge that the sample size constitutes an important limitation.

**Reviewer** **#2**

**Comment 18.** “This study provides valuable insights into the relationship between hearing loss and emotional recognition. However, several points require attention for improvement..”

Response:

Thank you for your positive assessment.

**Comment 19.** “The authors should address how relying on air-conduction audiometry instead of comprehensive audiometric profiles may impact the reliability of their findings, as this is a minor deviation from the protocol”

Response:

We agree that this is a limitation that may affect the reliability of our findings. We have addressed this in the *limitations* subsection of the discussion, on page 46 .

**Comment 20**. “The reported effect size (η² = 0.02, f = 0.14) suggests the study is only powered to detect very small effects. With a sample size of N=70 participants, this raises concerns about the robustness of conclusions, especially in clinical contexts where effect sizes are typically larger (Cohen, 1988). Consequently, the findings may not generalize to the broader population of individuals with hearing loss, as significant effects in psychology and audiology often necessitate larger samples for reliable conclusions (Maxwell & Delaney, 2004).

1. Cohen J. Statistical power analysis for the behavioral sciences. 2nd ed. New Jersey, NJ: Lawrence Erlbaum Associates; 1988.

2. Maxwell SE, Delaney HD, Kelley K. Designing experiments and analyzing data: A model comparison perspective. Third edition / Scott E. Maxwell, Harold D. Delaney, and Ken Kelley. | New York,: Routledge; 2017.”

Response:

We acknowledge that the study suffers suffersfrom a lack of power to detect small effects. The initial target sample size initial target sample sizewould have allowed us to allowed us to obtain significance for small but not minimal (η² = 0.01) effects. However, due to the smaller sample size of 41 participants, there are concerns regarding the detectable effects.. A sensitivity analysis conducted later indicated that we had sufficient power to detect medium-sized effects, which enabled us to detect several of the effects of interest (in relation to our predictions), however, not all. As even small effects can be of interest, we believe future studies should aim to overcome this limitation by including larger samples, ideally more than 70 participants, to improve reliability and generalizability.

**Comment 21.** ”Why happiness improved specifically. Does this finding correlate with existing literature on speech perception, and could it be linked to particular frequency bands? Provide mechanistic insights or referencing previous research.”

Response:

Regarding the acoustic characteristics of happiness, we note that happiness was characterized by a particularly high pitch. A previous study (ref 9), which we now mention in the discussion (page 42 ), shows that the recognition of happiness in speech is associated with several frequency-related parameters, including a positive correlation with pitch.

**Comment 22.** “Why non-verbal improvements do not mirror those observed in verbal expressions. Could frequency-related parameters elucidate this difference? Discuss further”

Response:

The results of our study unfortunately do not provide reliable answers to these questions, and the limited number of recordings of nonverbal expressions do not allow us to analyze the relationship between accuracy for different emotions and acoustic differences. We describe various frequency-related characteristics of different emotions in the Results under the new subheader “*Acoustics characteristics of nonverbal emotion expressions”*. These indicate, for example, that many frequency-related parameters for the nonverbal expression of happiness for example are not distinct compared to other emotions (with particularly high or low values), while some frequency-related characteristics of those nonverbal emotions improved are more distinct. This does not show, however, that such characteristics were related to the improvements in their recognition. We mention a reference in the discussion on page 42 (ref 12), which shows that the recognition of the nonverbal expressions of anger and fear was correlated with pitch. As such improvement for the nonverbal expressions of fear (and the somewhat smaller improvement of anger) could be related to clearer perception of pitch.

**Comment 23.** “While the similar confusion patterns between groups are noteworthy, the implications of these findings (e.g., perceptual imprecision versus qualitative differences) should be discussed further, considering how altered pitch perception may influence these findings”

Response:

Thank you, we have now deepened the discussion of our findings regarding patterns of confusion further in relation to the acoustic results. See e.g. page 42, and page 44 .

**Comment 24.** “The repetition of results previously covered in the "Results" section dilutes clarity. Instead, the discussion should focus on how the findings align with or challenge previous similar studies”

Response:

Thank you. We reiterate results in the discussion to elaborate on our findings in relation to the predictions made in the introduction in order to to review how well the predictions were fulfilled.Additionally, we include discussions regarding how our study aligns with previous research on vocal emotion recognition and the effects of amplification in individuals with HL. We do this to provide the reader with at comprehensive understanding of our findings. Nevertheless, we have made general changes to the discussion to reduce redundance and ensure the discussion is less repetitive. We hope that the discussion will be more comprehensive, yet still accessible.

**Reviewer #3**

***Abstract***

**Comment 25.** “It may be beneficial to briefly define phrases such as "linear amplification" for readers unfamiliar with audiological terminology.”

Response:

Thank you, we agree that this may be beneficial. We have now added a brief definition in the abstract: “ ..,we examined the effects of mild-to-moderate hearing loss and linear amplification (similar amplification across sound levels)”. Further description of linear amplification and the specific formula we implemented is provided in the *Study design and procedure* subsection of the ***Methods section***.

**Comment 26.** “Incorporating precise statistics or effect sizes pertaining to enhancements in emotion recognition could enrich the findings and their importance”

Response:

Thank you for this suggestion we have added the following in the abstract:

The hearing loss group had poorer emotion recognition for both sentences (*F*(1,38)=15.24, *p* <0.001, η^2^_p_=0.29, without linear amplification, and *F*(1, 38)= 5.62, *p*=0.023, η^2^_p_=0.13, with linear amplification) and non-verbal expressions stimuli (*F*(1,38)= 25.18, *p* <0.001, η^2^_p_=0.40, without linear amplification, and *F*(1, 38)= 10.30, *p*=0.003, η^2^_p_=0.21, with linear amplification). However, linear amplification significantly improved the recognition of happiness, (p < 0.001) which is distinguished by frequency parameters, in the sentences. Fear (p = 0.004), but also anger (p = 0.004), were improved by linear amplification in the non-verbal material.

***Introduction***

**Comment 27.** “The flow could be improved with appropriate section organization. For instance, categorizing the sections on “Vocal Emotion Recognition” and “Effects of Hearing Loss” with specific subheadings could enhance readability, my suggestion is to add these subheadings to the introduction text accordingly, such as under “Vocal Emotion Recognition” section

• Importance of Vocal Emotion Recognition

• Acoustic Characteristics of Vocal Emotions

• The impact of auditory impairment on the perception of voice emotions

Furthermore, add this subheading under “Effects of Hearing Loss” section

• Prevalence of Hearing Loss”

Response:

Thank you for your suggestion. When revising the introduction we landed in the following structure:

- A short introduction to the subject of the article.
- Vocal emotion recognition
- *Acoustic characteristics* of vocal *emotions*
- Prevalence and causes of hearing loss
- Perceptual effects of peripheral hearing loss
- Hearing aids
- The impact of hearing loss on vocal emotion recognition

**Comment 28.** “Now, the introduction does not include information regarding the demographics of the participants in the earlier studies, it is important to briefly explain (in one paragraph) the demographic data (other age groups) of individuals who participated in prior studies. This will help to contextualize the findings and ensure that they are applicable to a wider population”

Response:

We have added information regarding the demographics, specifically age demographics, of participants in earlier studies on vocal emotion recognition in individuals with hearing loss on page 6.

It now reads:

“Vocal emotion recognition is reduced in individuals with hearing loss in general (30-34) including mild-to-moderate hearing loss (31-33). For example, poorer recognition for individuals with mild-to-severe hearing loss compared to individuals with normal hearing have been found in young and middle-aged individuals (ages 22-74, 31) and in middle-aged to older adults (50-90 years old; 31, 33).”

**Comment 29.** “There are some typo errors e.g., “different lower--level features”, “not yielded entirelyconsistent results”, (see for example 5-8 and 16-18),. hearing loss that inloves involves, Thisis likely related to, commonly usedto characterize”

Response:

Thank you for pointing out these errors. We have reviewed the text and corrected all typos that we could find.

***Materials and Methods***

**Comment 30.** “Revise Inclusion/Exclusion Criteria: Clearly differentiate between inclusion and exclusion criteria”

Response:

We have revised these and now present the inclusion and exclusion criteria in separate subsections on pages 10 and 11.

**Comment 31.** “The p-value should be written as p<0.001 rather than p<.001”

Response:

We have changed all p-values from the .00 format to the 0.00 format

**Comment 32.** “The sociodemographic data should be presented in the results section.”

Response:

We have moved the sociodemographic data to the results section

**Comment 33.** “Table 1 Description: Summarize Table 1 with a sentence that emphasizes the significant demographic differences before its presentation. Also, the table needs to be presented in the results section”

Response:

Table 1 has been moved to the Results section. It is now Table 2, and is preceded by the following information (pages 18-19):

“ Demographic data and hearing thresholds of the participants are presented in Table 2. The mean age of the hearing loss group (M=63, SD=8) was higher than for the normal hearing group (M=52, SD=8), *t*(39)=4.42, *p*<.001, *d* =1.38. Therefore, to control for the potential influence of age on group differences, age was included in all statistical analyses including group as a factor. The distribution of gender χ2=0.51, *p*=.48, and degrees of educational attainment, χ2=1.47, *p*=.69, did not differ between the two groups. Nor did they differ in estimated general cognitive ability (G), as measured through the Matrices subtest from WAIS-IV, *t*(39)=0.26, *p*=.79, *d* =0.08.”, *d* =0.08 .”

***Results***

**Comment 34.** “The structure of the result might be improved by having clearer subsections, for example, Descriptive Data, Recognition Accuracy, Effects of Hearing Loss, and "Effects of Amplification, with titles for each subsection. The reader would be better able to navigate the findings in a methodical manner if this was done”

Response:

Thank you for these suggestions. It was also suggested by reviewer 5, in line with the overall need to emphasize the acoustic results more that we should have a separate subsection titled “Acoustic differences between emotions”. As the demographic data and recognition accuracy data are also descriptive, we thought it would be consistent for each subheading to be more precise. As such the new subsections are: *Demographic data,* *Acoustic characteristics of emotions in sentences , Acoustic differences between emotions in sentences,* *Acoustic characteristics of nonverbal emotion expressions , Recognition accuracy, Effects of hearing loss, Effects of amplification, Differences in confusion matrices*

**Comment 35.** “In Tables 3a and 3b, the authors may merge the columns of acoustic feature and acoustic parameter”

Response:

Acoustic features refers to the three overarching features: Frequency, Energy/Amplitide/Intensity and Spectral balance found in the first column of the table, while Acourstic parameters refers to the specific acoustic parameters that are presented in the second coloumn of the table. Therefore, we will keep the formatting with unmerged cells.

**Comment 36.** “All figures need improvement regarding their quality”

Response:

We have remade the figures with increased resolution in accordance with the journal guidelines (600 dpi).

***Discussion***

**Comment 37.** “However, the authors may suggest:

“1. Future research should evaluate sensorineural hearing loss based on clinical diagnosis rather than self-reporting.”

Response:

Thank you for this suggestion. We have added this sentence in *Limitations* (page 46).

**Comment 38.** “Table 5 was not mentioned or referred to it in the discussion section”

Response:

Table 5 is now referred to in the discussion on page . Due to additions and restructuring of the text it is now Table 9.

***Conclusion***

**Comment 39.** “The conclusion that authors have composed is coherent and highlights the principal conclusions of your study; but it would be advantageous to adopt a more official tone, implement a little rearrangement for enhanced coherence, and incorporate a more prospective outlook. Here is a polished iteration:

1. Restructured the principal points to facilitate a more seamless transition from overarching findings to particular results and implications.”

Response:

Thank you for your suggestion. We have attempted to follow this structure.

2. “Incorporated a request for further research, enhancing the conclusion's forward-thinking nature and highlighting potential avenues that your work may inspire”

Response:

Thank you for these suggestions. We have included some requests for future research in the conclusions. Due to the expected brevity of the Conclusions section these are all presented briefly, while we elaborate more in the Discussion. On page 47, we write:

”Since vocal emotion recognition is important for social communication, it is important that patients and their communication partners are made aware of an increased risk of emotional miscommunication related to hearing loss. Furthermore, future studies may examine whether vocal emotion recognition in individuals with hearing loss can be improved through training.”

**Reviewer #4**

**Comment 40.** “It is a great work. The authors discussed the problem in detailed way. It is eye-opening piece of work”

Response:

Thank you very much for your kind comments.

**Reviewer #5**

***Summary of study***

**Comment 41.** In the current study, listeners with normal hearing and listeners with hearing loss heard sentences and nonverbal vocal expressions produced with 5 different emotions, and identified which emotion was presented. Listeners with HL were tested with and without hearing aid processing of stimuli, to create aided and unaided listening conditions. The authors also measured the frequency-, amplitude-, and spectrum-related characteristics of the two classes of stimuli, to explain some of the differences in listener performance. The results showed that listeners with hearing loss generally performed less accurately than listeners with normal hearing, with a larger difference when unaided (18-22 points' difference for the two types of material) than when aided (10 points' difference). The improvement in aided listening was mainly due to better recognition of "happy" in sentences, and "anger", "fear" and "interest" in nonverbal expressions. There isn't much discussion about the acoustics of different emotion categories, except to note that the acoustic distinction between emotions doesn't map well onto the perceptual distinction between emotions.

Response:

Thank you for all of your insightful comments that have made it possible to improve our manuscript. Most importantly, we have expanded on the discussion (see e.g. pages 41 and 42) and added analyses of the acoustics.

***Main comment:***

**Comment 42.** “There have been multiple studies on hearing loss, hearing aids and vocal emotion recognition, but none of them have included a detailed investigation into the acoustics of their stimuli to explain which cues listeners with hearing loss might be using and how they might contribute to poorer performance. ***The most promising (and novel) contribution of this study was the investigation into the acoustic characteristics of emotional speech using a comprehensive suite of measurements, and linking the acoustics to hearing loss and hearing aid processing. However, the paper didn't really follow through on this aspect.*** The authors highlighted acoustics in the registered protocol, as well as in the introduction and methods of the current paper. There was a detailed table of acoustic measurements for the two classes of stimuli, but almost ***no integration of the large amount of acoustics results with the behavioral results***. This was surprising given the authors' comments about frequency resolution being affected in sensorineural hearing loss and speculation about what cues hearing aids might be amplifying. The connection between acoustics and listeners' performance was limited to a note in the discussion about unpublished data and how the association wasn't as expected. In its current form, it's not clear what new contribution this paper brings to the literature, apart from a minor (and unexplained) finding that hearing aids help listeners to recognize happiness in spoken sentences. ***To help form a more coherent narrative, the authors need to: (a) strengthen the acoustics aspect to carry it through the paper, and (b) reconcile why they think looking at acoustics is helpful with the studies they cited that suggest the emotion recognition deficit isn't just (or even mainly) due to peripheral/sensory losses”***

Response:

After consideration of your comments we decided to attempt to reintroduce the approach which we outlined in the stage 1 registered report. This was implemented in the following ways:

1. We have generated components based on acoustic parameters from the acoustic features (frequency, amplitude, spectral balance), in sentences.
2. We describe these components for each emotion and make comparisons between emotions.
3. We test which components that contribute to distinguish between emotions, using nominal regression.
4. There is now more discussion of the behavioural results, including the patterns of confusion in relation to the acoustic characteristics of different emotion categories.

Regarding the relative importance of peripherial hearing loss or loss of sensitivity relative psychoacoustic abilities or cognitive processes for vocal emotion recognition difficulties in individuals with hearing loss, the acoustic analyses unfortunately do not provide relaible answers to such questions, however some results such as those regarding the acoustic dissmimilarity between happiness and surprise give some support for the idea that hearing sensitivity and psychoacoustic abilities only partly explain vocal emotion recognition in both individuals with and without hearing loss.

The biggest contribution to the question regarding the relative importance of hearing sensitivity versus other abilities comes from the results regarding the effects of linear amplification. If hearing sensitivity was of little importance in relation to other abilities we might expect no improvement from linear amplification, while if reduced hearing sensitivity was the predominant cause of vocal emotion recognition deficits we might expect greater improvement. As you mentioned, our findings regarding effects of linear amplification were not explained, and remain unexplained even though we present some acoustic characteristics of different emotions which may be relevant. This is a limitation of the present study, which could be overcome in future studies, for example by including a larger number of recordings for each emotion compared to the present study, particularly for nonverbal expressions, so that associations between acoustic parameters and recognition accuracy can be reliably examined.

***Abstract***

**Comment 43.** “"making this a high-priority research topic" -- the authors could include a brief note about why, to strengthen the rationale.”

Response:

We have added a brief note in the abstract:

“As emotion recognition is an essential ability that affects social interaction, and in extension can impact well-being, understanding vocal emotion recognition difficulties is a high priority research topic”

***Introduction***

**Comment 44.** “p.5: "The average threshold, which includes several of these frequencies, is the pure-tone-average (PTA)" -- the authors could specify which frequencies, instead of "several"; there's no definition later on in the methods for PTA-4”

Response:

The reason the frequencies are not specified in the introduction section is that different definitions of PTA include different frequencies. For clarity, we have now added the following on page 5 in the Introduction:

“The specific frequencies included in measuring PTA varies, but it is common to include the frequencies of 500, 1000, 2000, and 4000 Hz, which is referred to as PTA-4”

**Comment 45.** “p.6: "Several studies have found mild-to-moderate hearing loss is associated with deficits in vocal emotion recognition (19, 27-29, but also see 30-31 for different conclusions)." -- this statement is rather vague and could be misleading for readers, implying that peripheral losses lead to emotion recognition deficits; the authors could clarify instead if these studies are referring to changes in higher-order auditory processing, which would make sense in the context of the next sentence about hearing aids not helping. Do the other studies (30, 31) find no association between hearing loss (peripheral or otherwise) and emotion recognition deficits at all?”

Response:

Thank you for drawing our attention to this. We have rewritten the section on the effects of hearing loss on vocal emotion recognition and elaborated more on the issue of the causes of vocal emotion deficits. It now reads (pages 7-8):

“While the studies presented here show that individuals with hearing loss, including mild-to-moderate hearing loss, have poorer vocal emotion recognition, questions remain regarding its causes. For example, low frequency hearing loss contributed only marginally to emotion prosody recognition in individuals with mild-to-moderate hearing loss, while high frequency thresholds did not contribute at all (30). In addition, Goy et al. did not find a significant association between the PTA and emotion recognition in individuals with mild-to-moderate hearing loss (31). However, Legris et al., examining recognition of nonverbal emotion expressions, did find a significant negative association between PTA and recognition accuracy in this group (33). As such, the association between hearing sensitivity and vocal emotion recognition in individuals with hearing loss might differ between emotions in speech and nonverbal expressions. Further, the evidence so far shows that the use of hearing aids does not normalize vocal emotion expressions, neither in speech (31, 35), nor non-verbal emotion expressions (33). Goy et al (31) found small, non-significant, improvements in the recognition of some emotions, particularly fear, (31), and Legris et al. found overall improvements but with remaining significantly poorer recognition compared to normal hearing individuals (33). It is unclear how important different factors, such as reduced hearing sensitivity, deficits in psychoacoustic abilities, and changes in cognitive processing are for explaining vocal emotion recognition deficits in individuals with mild-to-moderate hearing (32).”

***Aims***

**Comment 46.** “The first sentence is redundant, given the more specific aims that follow”

Response:

We agree with this and have therefore removed the first sentence.

**Comment 47.** “There is some inconsistency between the content of the introduction and the current study's predictions. For example: p.6: "Modern hearing aids use non-linear amplification and compression, which involves the selective amplification of more quiet sounds" -- the authors don't explain why they chose the Cambridge linear amplification formula, either in the Study Design or earlier”

Response:

The rationale for examining linear amplification is now presented on page 8 :

“Several aspects of hearing aid signal processing may influence vocal emotion recognition. In the current study, the audibility of the sound provided by hearing aid amplification is investigated. This is achieved through linear amplification based on individuals’ hearing thresholds using the Cambridge formula (36). The Cambridge formula aims to restore loudness perception across the entire speech spectrum for individuals with hearing impairment”We selected the Cambridge formula for linear amplification due to its established use in previous research, which provides a basis for comparison and validation of our findings. The Cambridge formula has been shown to effectively simulate real-world hearing aid performance. By using this formula, we aimed to ensure consistency with existing literature.

**Comment 48.** “p.6: "Hearing aids using linear amplification ... do not restore frequency selectivity", but prediction #5 says "The more salient the frequency-related acoustic parameters of an emotion are, the better that emotion will be identified when linear amplification is used"

Response:

We agree that this can appear inconsistent and needs some justification. It is true that a sensorineural hearing loss leads to decreased frequency selectivity. However, improving the audibility of the high-frequency content of the speech signal, that is often inaudible for the hearing impaired person, may improve the ability to resolve frequency content in the signal. This ability is further enhanced by the distinctiviness (saliency) of the acoustic frequency parameter.

***Methods***

**Comment 49.** “Figure 1 (audiogram) - a standard audiogram usually has the y-axis reversed”

Response:

We have changed the audiogram figure, reversing the y-axis.

**Comment 50.** “There could be a little more description of the nonverbal utterances, besides their duration. Perhaps a pseudo-phonetic example (e.g. "hmm") for each emotion could be provided?”

Response:

This is an interesting suggestion which we have considered. However, we found it difficult to describe and differentiate between some expressions in this manner. We found the best solution was to provide a link in the text to where the material is stored on the Open Science framework. As such anyone can listen to the nonverbal expressions if they wish.

**Comment 51.** “p.12 "The stimulus material is based on fourteen emotionally neutral sentences from the Swedish version of the hearing in noise test (HINT; 38), and non-verbal emotion expressions." -- It would be clearer to mention there are 20 nonverbal stimuli here, instead of presenting that information on p.15”

Response:

Since there are some differences between these stimulus types, such that all of the 14 different sentences were expressed for all emotions we thought it better to give more detailed information before information about the number of recording per stimulus type.

**Comment 52.** “p.12: It might also be easier for the reader to keep track of the emotion categories that were used, if there was a brief note on p.12 about which categories of emotions were dropped, instead of that information only appearing in the validation section later on p.13.”

Response:

We now describe which emotion categories were dropped, and which were included in the study in the *Stimuli material* section (pages 12-13.).

**Comment 53.** “p.15 "Thus, there were in total 108 sentences (x with anger, y with happiness, z with fear, v with sadness, and w with surprise) and 20 non-verbal expressions (a with anger, b with happiness, c with fear, d with sadness, and e with interest)." -- the authors need to fill in the numbers”

Response:

We apologize for this oversight. We have now filled in the correct numbers on page 15.

**Comment 54.** The authors could specify if the acoustics are mean-centered (not just centered). Were they normally distributed, if that matters for the z-transformation?

Response:

We now write, on page 17: “To control for baseline parameter differences between speakers’ voices, z-scores were calculated for each parameter and for each sentence, using the means and standard deviations of each speaker’s neutral voice.“

Regarding normal distribution of the GeMaps parameters, we did not find any major deviations that prevented us from assuming normal distribution of parameters in the population.

**Comment 55.** Prosody was mentioned in th introduction as well as the registered protocol. Which of these GeMAPS measures would reflect prosody, and could they explain some of the difference in performance between the two hearing groups?

Response:

We do mention prosody as a form of vocal emotion expression in speech, contrasting it to nonverbal expressions. We did not intend to measure have any direct measures of prosody in this study examining the acoustic parameters of GeMaps instead as we specified that we would.

**Comment 56.** Table 2 -- instead of leaving F0 a blank, perhaps the authors could note how F0 is calculated in GeMAPS (e.g. cross-correlation, etc)

Response:

Thank you for noticing. “F0” has been removed from the table since it represents the same as Pitch in GeMAPS..

**Comment 57.** What software was used for the statistical analysis? The authors could also specify which factors were between- or within-subjects, just to be clear about the different types of group comparisons.

Response:

We now write under statistical analyses, on page 19: “The statistical analyses were performed in IBM SPSS v. 29.0.2.0”

Further, we now describe which factors were between-or within subjects analyses on page :

**Comment 58.** “p.18 Rosenthal's Proportion Index - it wasn't immediately clear what this note referred to, as it appeared in the section on accuracy/behavioral analysis. It would be better to move this to the earlier section on acoustic analysis since it's relevant to the stimuli - if it does refer to the acoustic measurements? A brief explanation about what effect size the index refers to would be helpful, e.g. differences between emotions.”

Response:

Rosenthals pi is an effect size estimator useful for comparisons across studies with different number of response options. Since it refers to the response data, not the stimuli, it needs to be reported together with the accuracy data. However, we have added a description on page 18 to clarify the use of the measurement:

” Rosenthal’s PI is a measurement of the accuracy rate for response data which spans between 0.5 and 1 and is calculated based on the obtained rate in relation to the number of response options. A specific accuracy rate for a response category will be calculated as higher when there are more response options available and lower if there are fewer response options (47).”

***Results***

**Comment 59.** “In general, the results section could be better organized. If the authors are mainly interested in the effects of hearing loss and hearing aids, the results could be organized according to these sections, instead of separating them by the class of stimuli (verbal and nonverbal). It would also make it easier to spot overall trends across classes of stimuli.”

Response:

Thank you for your suggestion, we have now used the following structure of the results section:

- *Demographic data*
- *Acoustic characteristics of emotions in sentences*
- *Acoustic differences between emotions in sentences*
- *Acoustic characteristics of nonverbal emotion expressions*
- *Recognition accuracy*
- *Effects of hearing loss*
- *Effects of amplification*
- *Differences in confusion matrices*

**Comment 60.** “The behavioral results may be easier to follow with tables for the main effects and interactions and associated numbers, and a narrative summary of the overall pattern of results”

Response:
Thank you for these suggestions. We have added a table with summaries of all main effects and interactions in supporting information, “SI Table and narrative summaries of the overall patterns of results on pages 34-36 and 37-38.

**Comment 61.** More appropriate sub-headers could be used; e.g., instead of "Descriptive data" (p.18), it could be "Acoustic differences between emotions" or something like that. There is also no description of the acoustic findings for nonverbal stimuli besides the fact that they differ from the sentence stimuli.

Response:

See response 59 for the new structure of the results section. We have also added descriptions of the findings for nonverbal expressions.

**Comment 62.** “p.17 "The same analyses were then repeated but using sessions with amplification for the hearing loss group." -- could be rephrased to improve clarity; also, the unaided to aided comparison wouldn't be a between-groups analysis but rather within-groups”

Response:

We have attempted to clarify this by writing: “Participants with hearing loss performed a total of four sessions; two sessions with linear amplification (aided listening condition), one for sentences and one for non-verbal expressions, and two sessions without linear amplification (unaided listening condition), one for sentences and one for non-verbal expressions.”

**Comment 63.** “I suggest being consistent about capitalizing main and interaction effects.”

Response:

Thank you for noticing this inconsistency. We have now capitalized all the effects.

**Comment 64.** “Table 4 - typo in "Rosenthal". Also, a brief note in the caption about what Rosenthal's Index refers to here would be helpful, e.g. difference between which groups. Are there different versions of the index for within-group (one sample) and between-group (two samples), if this is relevant?”

Response:

Thank you for pointing out the typo, we have fixed it. We have added an explanation of what Rosenthals proportion accuracy measures on page 18.

**Comment 65.** “p.28 "Difference in confusion matrices" -- these percentage values appear to refer only to one direction of Expressed --> Identified cells in the matrix, e.g. "happy" identified as "surprised". But wouldn't confusion of two emotions go both ways, so that the measure of confusion should incorporate both "happy" --> "surprised" and "surprised" --> "happy"?”

Response:

The information that you request is presented in the table. If you seek information about how frequently happiness is confused with surprise this is presented in the row for happiness and column for surprise, and if you wish to know how frequently surprise is confused with happiness this is presented in the row for surprise and column for happiness. We used his structure for confusion matrices since it is to the best of our knowledge standard in behavioural research.

**Comment 66.** “Table 3A -- can the Pitch measure be 5 to 7 SD above the mean for neutral? Is Pitch a more standard measure in the emotion acoustics literature than F0?”

Response:

We agree that this might seem surprisingly high, but this can happen for example if acoustic parameters for an emotion are much larger than in the reference of neutral speech. This can also be facilitated by small standard deviations in neutral speech. Pitch, as calculated in GeMaps, is F0 measured on a logarithmic scale.

**Comment 67.** Which of these GeMAPS measure capture prosody?

Response:

None of these are a direct measurement of prosody.

***Discussion***

**Comment 68.** “p.29: "we found patterns of confusion to be similar for both groups and regardless of amplification, which suggests that the two groups perceived different emotions similarly, but that the degree of perceptual precision was lower for individuals with hearing loss." -- this statement doesn't really "suggest" anything. Rather, it shows results, which need interpretation to suggest something.”

Response:

We agree that this phrasing is incorrect and have changed it to:

“We interpret this as suggestings that mild-to-moderate hearing loss and linear amplification in relation to vocal emotion recognition are mostly associated with differences in degrees of perceptual precision, rather than qualitative perceptual differences”

**Comment 69.** “It might help to orient the reader if predictions were in the same order as before; e.g. Prediction 3 is presented last now”

Response:

We chose to rearrange the predictions in the Introduction to match the order in the Discussion instead, as we find that the order in the discussion iseasier to follow logically.

**Comment 70**. “The authors grouped the acoustic measurements into frequency-, amplitude- and spectral-related measurements earlier, and also mentioned that sensorineural hearing loss and hearing aid processing might affect different characteristics differently. What happened to this chain of thought?”

Response:

We have reintroduced the analysis of which overall feature distinguish different emotion categories in the Results section and attempt to relate findings to the predictions we made, in the discussion. We also relate these findings in sentences to the patterns of confusion in the discussion.

**Comment 71.** “p.31: "in the present study, recognition of happiness was poorer for the hearing loss group, and in the hearing loss group it was also, in contrast to the normal hearing group, poorer than recognition of anger and sadness. In summary, results were indicative, but not substantial, for the second part of prediction 4." -- needs some rephrasing to improve clarity”

Response:

During the revision or the discussion, this text has been removed. The discussion of this prediction (now the second part of prediction number 3) is now made in light of findings regarding which acoustic features distinguish different emotions, rather than which contribute to recognition.

***Limitations***

**Comment 72.** "Most people can be expected to be aware of their type of hearing loss" - I'm not sure that most people would know whether or not they have sensorineural hearing loss... Can the authors use their answers from their screening; e.g. reported noise exposure through occupation or leisure activities, ototoxic medications, self-reported past hearing problems etc?”

Response:

We agree and have rephrased and extended this part of the limitations. In that, we also suggest that future research should (if possible) determine the type of hearing loss based on clinical diagnosis.

The question to participants in the survey was whether the cause of their hearing loss was conductive which included multiple common causes of conductive hearing loss). Based on the negative responses to this question we think it is reasonable to conclude that the cause of participants hearing loss was not conductive problems. This survey question was not previously described in Participants but has now been added under *Exclusion criteria*.

We have also added the following as an argument in favour of classifying participants as having sensorineural hearing loss on page 46:

“It is important to note, however, that the participants’ audiograms showed good low-frequency thresholds, suggesting that conductive losses did not significantly affect their hearing. Additionally, their audiograms showed a pattern of sloping high-frequency hearing loss, indicating a sensorineural hearing loss of presbyacusis origin”

***Conclusions***

**Comment 73.** “Table 5 should probably be located earlier, in the discussion section on predictions.”

Response:

It has now been located to earlier in the discussion and has number 9.
